# Palmitic Acid Induces Dynamic Time-Dependent Alterations in HDACs, Neuronal Chromatin Acetylation, and Gene Expression

**DOI:** 10.1007/s11064-025-04469-w

**Published:** 2025-06-30

**Authors:** Saúl Santiago Rueda-Díaz, Iker Francisco Soto-Santarriaga, Karla Torres-Arciga, Valeria Melissa García-Cruz, Rodrigo González-Barrios, Manuel Flores-León, Clorinda Arias

**Affiliations:** 1 Departamento de Medicina Genómica y Toxicología Ambiental. Instituto de Investigaciones Biomédicas, Universidad Nacional Autónoma de México (UNAM), AP 70-228, Ciudad de México, 04510 México; 2 Department of Chemistry & Biochemistry, University of Notre Dame, Notre Dame, IN 46556 USA; 3 Unidad de Investigación Biomédica en Cáncer, Instituto de Investigaciones Biomédicas (IIB), Instituto Nacional de Cancerología (INCan), Universidad Nacional Autónoma de México (UNAM), Ciudad de México, 14080 México; 4 Department of Experimental Neurodegeneration, Center for Biostructural Imaging of Neurodegeneration, University Medical Center Göttingen, Göttingen, Germany

**Keywords:** High fat diets, Palmitic acid, HDACs, Histone acetylation, *BDNF*

## Abstract

Chronic consumption of high fat diets (HFD) is a risk factor for the development of metabolic diseases such as obesity and diabetes, and it is also associated with cognitive impairment and Alzheimer´s disease. Palmitic acid (PA) is a major component of HFD, and high concentrations of this saturated fatty acid exerts pleiotropic actions in cells. The PA effects have been largely studied in peripheral tissues where is considered a driving force for the development of many metabolic diseases such as obesity, insulin resistance and Type II diabetes. In the brain, particularly in neurons, it is able to increase oxidative metabolism, induce insulin resistance, and alter gene expression. However, little is known about how PA-induced metabolic alterations may affect gene expression mechanisms in neurons. One of the most studied PA-dependent mechanisms is associated with the lipid-induced activation of the transcription factors, PPAR-γ and PGC-α, but fewer studies have analyzed the PA-dependent regulation of epigenetic mechanisms. In this study, we identified PA-linked changes in the class I histone deacetylases (HDACs) content associated with chromatin acetylation and with differential expression of the *BDNF-*encoding gene and the non-coding retrotransposon, *LINE1* in differentiated human neuroblastoma cells.

## Introduction

Saturated fatty acids (SFA) are the main component of high-fat diets (HFDs), whose high consumption is associated with adverse health effects such as obesity, insulin resistance, Type II diabetes, and metabolic syndrome [1–3]. The chronic consumption of HFD is considered a significant risk factor for cognitive decline, pathological brain aging, and even Alzheimer´s disease (AD) [4–8]. Palmitic acid (PA) is the most abundant SFA found in HFD and its importance for metabolic alterations in the central nervous system has recently been recognized. Different research groups have provided strong evidence of the contribution of PA in neuronal energy metabolism [9, 10], reduced insulin sensitivity [10–12], increased ceramide production and neuroinflammation [13–15], and reduced neuronal viability [16]. In this context, HFD intake significantly decreases the NAD^+^/NADH ratio in liver [17]. Similarly, we have reported that after 24 h of PA overload, there was a reduction in the histone deacetylase (HDAC) Sirtuin 1 (Sirt1) associated with NAD^+^ depletion along with changes in the transcriptome profile in hippocampal neurons [18]. Hence, neurons exposed to a high concentration of PA appear to initiate critical metabolic rearrangements that in turn have repercussions on gene transcription. Gene expression is regulated by epigenetic mechanisms that change the chromatin state and structure [19], thus allowing genes to dynamically adapt to internal and external stimuli. These changes in the epigenome can be driven by drug consumption, physical activity, stress, and, relevant for this study, diet and its components [20, 21]. In this regard, several studies in rodents exposed to HFD demonstrate some changes in cellular mechanisms that may lead to an altered function of the epigenetic machinery, such as hypermethylation of the transcription factor PGC-1α [22] and, through the increase in the acetyl-CoA pool generated by the β-oxidation of fatty acids.

Therefore, we investigated whether neuronal exposure to PA induces changes in HDACs that may contribute to the previously observed transcriptional deregulation. Specifically, we aimed to assess the temporal effects of PA exposure in the content of class I deacetylases, HDAC2 and HDAC3. Additionally, we examined whether these changes correlated with chromatin acetylation and with the transcriptional regulation of the protein coding gene, Brain-derived neurotrophic factor (*BDNF)* and the non-coding gene, Long interspersed element-1 (*LINE1)* in a differentiated human neuroblastoma cell model.

## Materials and Methods

### Cell Culture

Human neuroblastoma cells (MSNs) (SMS-MSN cells, RRID: CVCL_7135) [23] were kindly provided by Dr. R. Gutiérrez at Albert Einstein College of Medicine. The cells were seeded for protein and RNA extraction or immunofluorescence assays at a density of 2.5 × 10^5^ or 1.5 × 10^5^cells per 35 mm well, respectively, and maintained in RPMI 1640 (Gibco©) supplemented with nonessential amino acids (REF: 11140-050, Gibco©), 10% fetal bovine serum (FBS) (Gibco Invitrogen, Grand Island, CA) in an atmosphere with a saturation of 5% CO2/95% O2 at 37 °C. Once they had adhered to plates and reached a 50% confluence rate, cells were differentiated into a mature neuron-like phenotype following a modified protocol previously established [24–26]. This protocol yields homogeneous populations of neuronal cultures that exhibit many characteristics of mature neurons [27] such as long and extensively branched neurites and the expression of mature neuron’s markers such as the microtube-associated protein 2, tubulin β-III, and an increased expression of the cell cycle inhibitor p21 protein [24, 28, 29].

This consisted in the replacement of the original medium with differentiation medium, RPMI supplemented with retinoic acid (10 µM) and nerve growth factor (NGF) (50 ng/mL) and subsequent culturing for 5 days. On the fourth day, half of the medium was replaced with fresh differentiation medium.

### Palmitic Acid Treatment

PA (Sigma-Aldrich, USA) was prepared as an initial 200 mM stock solution in absolute ethanol (Sigma-Aldrich). Out of this stock solution, a 5 mM working solution was freshly prepared on the day of each experiment using a sterile solution of PBS-BSA 10%. BSA was used as a carrier protein to allow the fatty acid to remain in solution. This solution was then incubated at 37 °C for at least 1 h with occasional gentle shaking. Once the PA was completely conjugated with the PBS-BSA 10% solution, it was added to the cell cultures at a final concentration of 300 µM for 3, 6, 12–24 h. Ethanol 2.5% in PBS-BSA 10% was used as vehicle and as the control condition for a final concentration of 0.15% in culture.

### Trypan Blue Viability Assay

Viability Assays were performed using the Trypan Blue exclusion cell counting. Once differentiated, 3 concentrations of PA were added to the cell cultures (300, 400 and 500 µM) for 24 h. After the PA treatment, a PBS wash was performed and immediately the cells were harvested using 200 µL of trypsin and incubated at 37 °C for 5 min. Trypsin was inactivated after 5 min by the addition of 300 µL of differentiation medium. Cells were collected and centrifuged at 1500 rpm for 3 min. Supernatant was discarded and the cell pellet was gently resuspended in differentiation medium.

A working solution was prepared by adding in equal parts of cell sample and Trypan Blue (1:1), and it was softly resuspended. Next, 12.5 µL were pipetted into a Neubauer chamber. Live and dead cells were counted with the aid of an optical microscope, where cells dyed in blue were considered dead and cells with no dye and light-refractory were considered alive. Finally, the percentage of live and dead cells was calculated by comparing the number of each against the total number of cells.

### Beta-hydroxybutyrate (β-HB) Quantification

β-HB was quantified using a Colorimetric Assay Kit (Cayman Chemical, 700190) according to the manufacturer´s instructions. Briefly, after a 24 h treatment with PA (200 or 300 µM) cells were harvested with the assistance of cell scrapers. For each condition (PA-treated or vehicle-treated), 18 × 10^6^ cells were collected. These cells were centrifuged at 1,500 g for 10 min. After this, cell pellet was resuspended in assay buffer (Tris-HCl 0.1 M, pH = 8.5). This cell suspension was sonicated and subsequently centrifuged at 10,000 g for 10 min at 4 °C. Quantification was performed by the addition of 50 µL of cell samples and 50 µL of developer solution in a microplate, which was incubated for 30 min. Next, absorbance was measured at 450 nm using the plate reader Epoch (BioTek Instruments, Inc). The data analysis was performed by generating a line of best fit to obtain the equation of the line based on the concentration and absorbance of the samples of the standard curve. Then, the average of the absorbance readings of the replicates of each sample was averaged and this value was substituted into the straight-line equation to obtain the concentration of each sample.

### Oil Red Staining and Quantification

The detection of lipid droplets was carried out using the oil red staining, Oil Red O (Sigma, O0635). Briefly, Oil Red O stock solution is prepared by adding 5% Oil Red O in isopropanol 100% at constant stirring overnight at 4 °C. Then, the solution was filtered using a Whatmann filter paper. A working solution was prepared in water at a 6:4 ratio and incubated at room temperature (RT) until its immediate use. The working solution was filtered with a 0.2-micron filter. Then, the culture media was removed, and cells were washed and fixed during 1 h with PFA 4% in PBS. The fixing solution was removed, followed by a PBS wash and a 60% isopropanol wash. After removing the isopropanol, the cells were left to dry, and the oil red working solution was added for 2 h at RT. Finally, the oil red staining solution was removed, and the cells were washed 3 times with distilled water or until the red background from the plate disappeared. Cells were observed under an inverted fluorescence microscope (Olympus IX71) to detect the presence of lipid accumulation. To perform the colorimetric assay, 100% isopropanol was added to the cells for 20 min with gentle agitation. 200 µL were taken from the culture wells and measured at 520 nm in a 96-well plate using a spectrophotometer (BioRad Microplate reader 550).

### Protein Quantification by Western Blotting

Mature human differentiated neuroblastoma cells were homogenized at 4 °C in RIPA lysis buffer (50 mM Tris pH 7.5, 150 mM NaCl, 0.5% sodium deoxycholate, and 1% Nonident P40, protease inhibitor cocktail (Roche Diagnostics). Protein concentration was measured using a BioRad DC protein assay kit (500 − 0112) and the plate reader Epoch. Equal amount of protein (30 µg) were boiled with Laemmli buffer (TRIS-HCl 0.5 M pH = 6.8, SDS 10%, glycerol, blue bromophenol 0.25%) and then separated by electrophoresis on acrylamide-SDS gel. The proteins were transferred to a PVDF membrane (REF: IPVH00010, Millipore) and blocked with 5% BSA in Tris-/NaCl/Tween-20 (TBS-T 0.1%) for 1 h at RT. After that, membranes were incubated overnight at 4 °C with the following primary anti-bodies: SIRT1 (1: 2000, Abcam, ab104833), HDAC3 (1:1000, Santa Cruz Biotechnology, SC-11417), HDAC2 (1:1000, Santa Cruz Biotechnology, SC-9959), H3 Total (1:2000, Abcam, ab18521), H3K9ac (1:1000, Active Motif, 39137) and β-actin (1:1000, Sigma, A5316). At the end of this period the membranes were washed 3 times with TBS-T and incubated for 2 h at RT with the following secondary antibodies at 1:10000 dilution: rabbit anti-mouse antibody (Invitrogen, 61-6520) or goat anti-rabbit antibody (Invitrogen, 31460). Membrane signals were revealed using a chemiluminescent ECL substrate (Millipore, WBKLS0500) on Kodak X-Omat films. A densitometry analysis of the bands was performed using ImageJ software (NIH).

### Immunofluorescence and Image Analysis

After PA treatments, the cell culture media was removed, and cells were washed three times with ice-cold PBS. Then, the cells were fixed with ice-cold PFA 1%/PBS for 10 min and washed three times with PBS for 5 min. Afterwards, cells were permeabilized in PBS/Triton 0.3% for 20 min at RT and incubated in a blocking solution (PBS/Triton 0.3%/BSA 5%) with gentle agitation for 1 h at RT. Next, cells were incubated overnight with anti-H3K9ac antibody (1:2000, Active Motif, 39137) diluted in blocking solution at 4 °C. After washing three times, 5 min each, in PBS/Triton 0.3%, cells were incubated with the secondary antibody (1:2000, Alexa Fluor 488 donkey anti-rabbit, Invitrogen, A21202) for 2 h at RT. Immediately, nuclei were stained with DAPI (1:700) in PBS for 10 min at RT and mild agitation. Cells were washed three times with PBS and covered with a fluorescent mounting medium (DAKO). Negative controls were performed excluding the primary antibodies from the procedure. Observations were performed on a Nikon A1R + confocal microscope (Nikon Instruments Inc.) with a Plan Apo 40 × (N.A. 0.95) objective and digital images were obtained with the NIS-Elements C imaging software (Nikon). Following image acquisition, 100 randomly selected cell nuclei per condition were analyzed to evaluate H3K9ac intensity and distribution. Z-stack images consisting of 25 focal planes were captured with 0.6 microns between each focal plane. Analysis was conducted using Fiji© software. Maximum intensity projection was applied for intensity quantification, and the Rainbow RGB colorimetric filter was used to assess signal distribution.

### RNA Extraction and Quantitative RT-PCR

Total RNA was isolated using TRIzol reagent (Thermo Fisher Scientific) as specified by the manufacturer. Briefly, 500 µL of TRIzol was added to each well, cells were scrapped, collected and frozen for at least 24 h. Next, cells were centrifuged, and supernatant was transferred to a new tube and chloroform was added for 5 min at RT. After centrifugation aqueous phase was separated in a new tube, mixed with isopropanol, incubated for 10 min at RT, and centrifuged once again. The pellet was softly washed with pure ethanol and left to dry. Finally, the pellet was diluted with RNase-free water and incubated at 55 °C for 10 min. After RNA isolation, RNA integrity was assessed by a 1.2% Agarose-Gel Electrophoresis using GelRED (Biotium, 41003) in denaturing conditions with 4 M urea buffer. Samples were prepared with a mixture 1:1 of the 4 M urea and the loading buffer 10x (blue bromophenol 0.25%, EDTA 0.5 M, SDS 10% and glycerol) and were heated up to 55 °C for 10 min. Samples were loaded to the agarose gel and an electrophoresis was performed at 120 V. Images were captured using a Kodak Gel Logic 200 Imaging System© photodocumenter with UV/Vis light. RNA concentration and purity was measured by using the plate reader (REF: EPOCHH, Serial Number: 1412137, BioTek Instruments, Inc©) in the “Nucleid Acid Quantification” modality. Each sample was measured in duplicate. The cDNA was synthesized from 1000 ng of RNA using the Kit ImProm-IITM Reverse Transcription System (Promega, A3800) and quantified in the plate reader. RT-qPCR was performed using the Maxima SYBR Green/ROX qPCR Master Mix (Thermo Scientific, K0221) in a Step One real-time PCR system (Applied Biosystems). Gene expression was normalized using *glyceraldehyde-3-phosphate dehydrogenase* (*Gapdh*) as an endogenous gene. Data analysis was performed using the ΔΔCT method. Primer sequences are as follows: For *BDNF* F: 5 ´- TTAAGCCTTTTCCTCCTGCT-3 ´ R: 5 ´- TCATCACTCTTCTCACCTGGT-3 ´. For *LINE1* F: 5´-GGCCAGTGTGTGTGCGCACCG-3´ R: 5´-CCAGGTGTGGGATATAGTCTCGTGG-3´. As for *GAPDH F: 5 ´- CACTCCTCCACCTTTGACG-3 ´ R: 5 ´- GTGGTCCAGGGGTCTTACT-3 ´*.

### Statistical Analysis

Statistical tests were chosen according to the number of samples and groups. All data are expressed as the mean ± standard error (SEM). Differences were considered statistically significant when *p* < 0.05. Statistical analyses were performed with Prism 9 software (9.0.1). The statistical test used to analyze the data obtained from the viability assay, lipid droplet and for ketone body quantification was a one-way ANOVA. For Western Blots, immunofluorescence analysis and RT-qPCRs, two-way ANOVA were performed. Šídák’s post hoc test was used to evaluate the statistical results. Control data are depicted as the percentage of the mean of all control experiments for each experimental condition.

## Results

### PA Exposure Induces β-HB Production and Lipid Droplet Accumulation

**Fig. 1:**
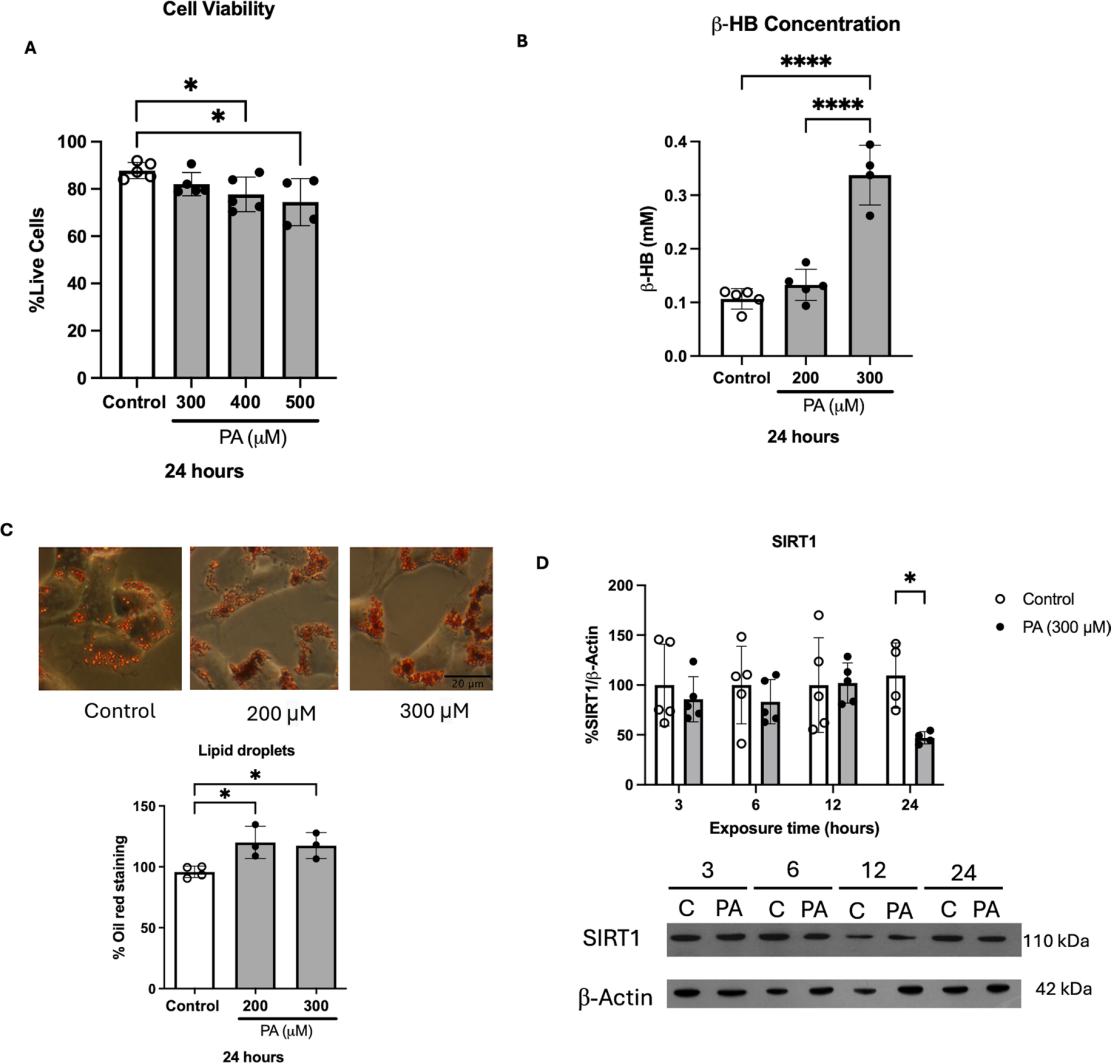
Metabolism of PA in neurons. (**A**) Cell viability evaluation by trypan blue assay. No significant differences are observed between the neurons exposed to 300 µM PA and the control group. Cytotoxic effects when the neurons are exposed to 400 µM (*p = 0.0394) and 500 µM (*p = 0.0166) PA. Analyzed by one-way ANOVA (*n* ≥ 4). Results shown are the mean ± S.E.M. of 4–5 independent experiments. (**B**) Quantification of β-HB concentration. A 216% increase in β-HB concentration was observed after 24 h of 300 µM PA treatment with respect to the control group and a 154% increase respect with the group treated with 200 µM PA (*n* ≥ 4, ****p < 0.0001). Data was analyzed by one-way ANOVA (*n* ≥ 4). (**C**) Lipid droplets quantification. An increase in the accumulation of lipid droplets was demonstrated by oil red staining after a 200 and 300 µM treatment for 24 h. Analyzed by one-way ANOVA (*n* ≥ 3). Results shown are the mean ± S.E.M. of 3–4 independent experiments. (**D**) SIRT1 protein content quantification demonstrates a decrease after 24 h of PA exposure (*n* ≥ 4, *p = 0.0384). Analyzed by 2-way ANOVA. Results shown are the mean ± S.E.M. of 4–5 independent experiments

First, to determine that the concentration of 300 µM of PA, found in the serum of obese and diabetic patients [30, 31], had no cytotoxic effects on differentiated neuroblastoma cells, a trypan blue viability assay was performed after a 24-hour treatment with PA concentrations up to 500 µM (Fig. 1A). Treatment with 300 µM PA induced a reduction of less than 10% in cell viability, compared to the control group. However, with higher concentration of 400 and 500 µM a significant reduction in cell viability was observed. This is consistent with previous findings, where the MTT assay indicated no cytotoxicity at concentrations up to 200 µM after a 24-hour PA treatment while the trypan blue assay detected cytotoxic effects only after 500 µM PA treatment [24]. We then investigated whether PA, at concentrations up to 300 µM, can be metabolized and stored in neurons by measuring the production of the ketone body β-HB and the presence of lipid droplets. We found that after 24 h of exposure, only the 300 µM of PA condition led to a significant increase in β-HB production (Fig. 1B). Additionally, both PA concentrations resulted in a 20% increase in neuronal lipid droplet content (Fig. 1C). Considering that another long-term reported effect of PA on cellular metabolism is the reduction of the content and activity of the metabolic sensor SIRT1 [32], we analyzed the temporal course of changes in SIRT1 protein content after 3, 6, 12, and 24 h of PA. Our data showed that SIRT1 remained unchanged at short incubation periods (3, 6, and 12 h) but decreased after prolonged exposure to PA (24 h) (Fig. 1D).

These findings indicate that PA is metabolized in neurons through different pathways and that a non-cytotoxic PA concentration, such as 300 µM, promotes lipid accumulation and energy metabolism activation. Based on these results, we conducted all subsequent studies using this concentration of PA.

### Differential Time-Dependent Changes in HDAC2 and HDAC3 After PA Exposure

**Fig. 2:**
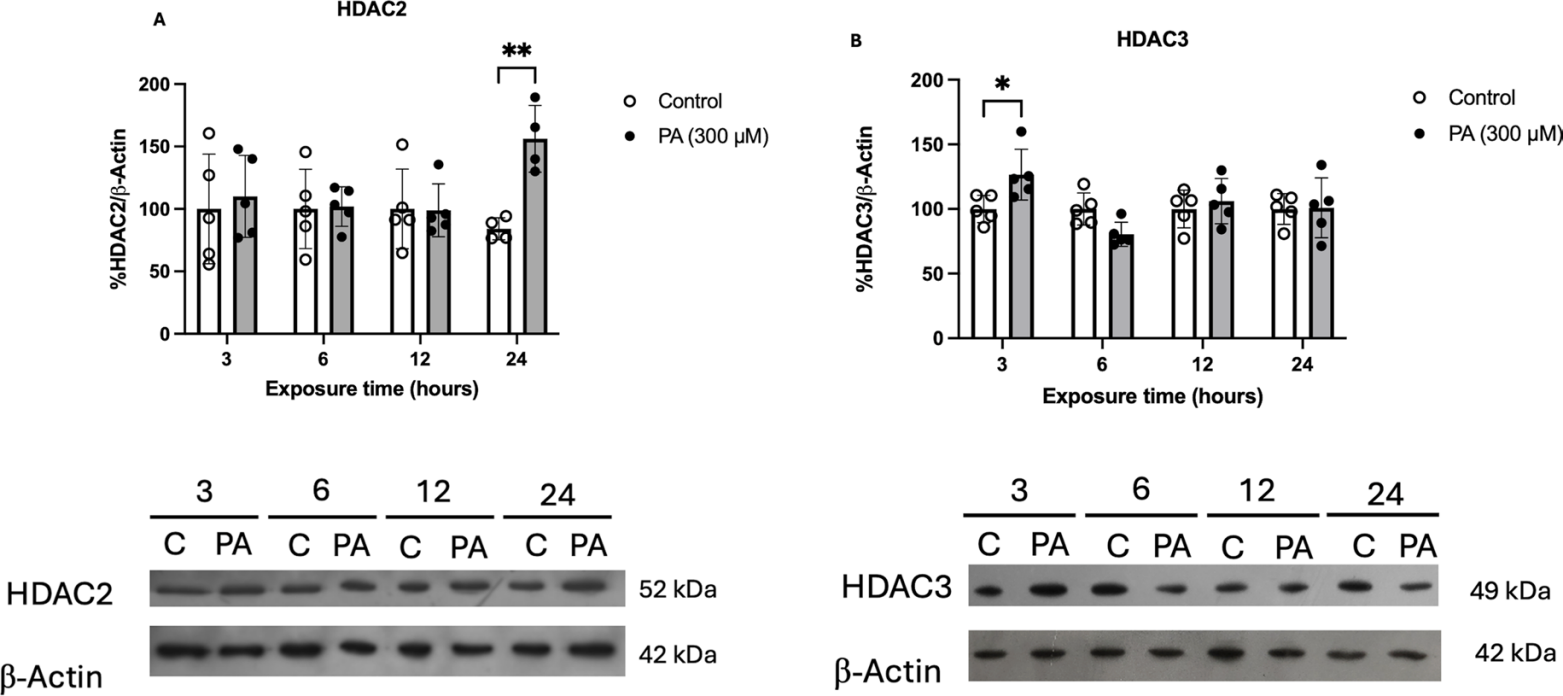
Differential time-dependent increase in HDAC2 and HDAC3 protein content after PA exposure. (**A**) Densitometric analysis and representative western blot of HDAC2. Quantification shows that HDAC2 increases after 24 h of PA treatment (**p = 0.0057). (**B**) Densitometric analysis and representative western blot of HDAC3. Conversely, increase of HDAC3 was observed after 3 h of PA exposure (*p = 0.0108). Analyzed by 2-way ANOVA (*n* ≥ 4). Results shown are the mean ± S.E.M. of 4–5 independent experiments

We next analyzed if HDACs (2 and 3) were affected after neuronal exposure to PA which may be a possible link between this saturated fatty acid metabolism and altered gene expression. Due to their nuclear enrichment and the high affinity of class I HDACs to acetylated histones [33], we measured the content of HDAC2 and HDAC3 at different exposure times of PA (3, 6, 12, and 24 h) (Fig. 2). The protein content of both HDACs followed different time-dependent dynamics after PA exposure. For HDAC2, the content remained constant until 24 h, when an increase was observed in response to PA treatment (Fig. 2A). Conversely, HDAC3 showed a short-term response as its content increased after 3 h of PA exposure to subsequently decrease to similar levels as those as the control condition (Fig. 2B).

These data indicate that HDAC2 and HDAC3 respond differentially and time-dependently to PA. As we found no significant differences of any HDAC expression after a 12-hour treatment, the following analyses were evaluated only at 3, 6 and 24 h of PA exposure.

### H3K9ac Increases its Content and Shifts its Nuclear Distribution Following a Short-Term PA Exposure

**Fig. 3:**
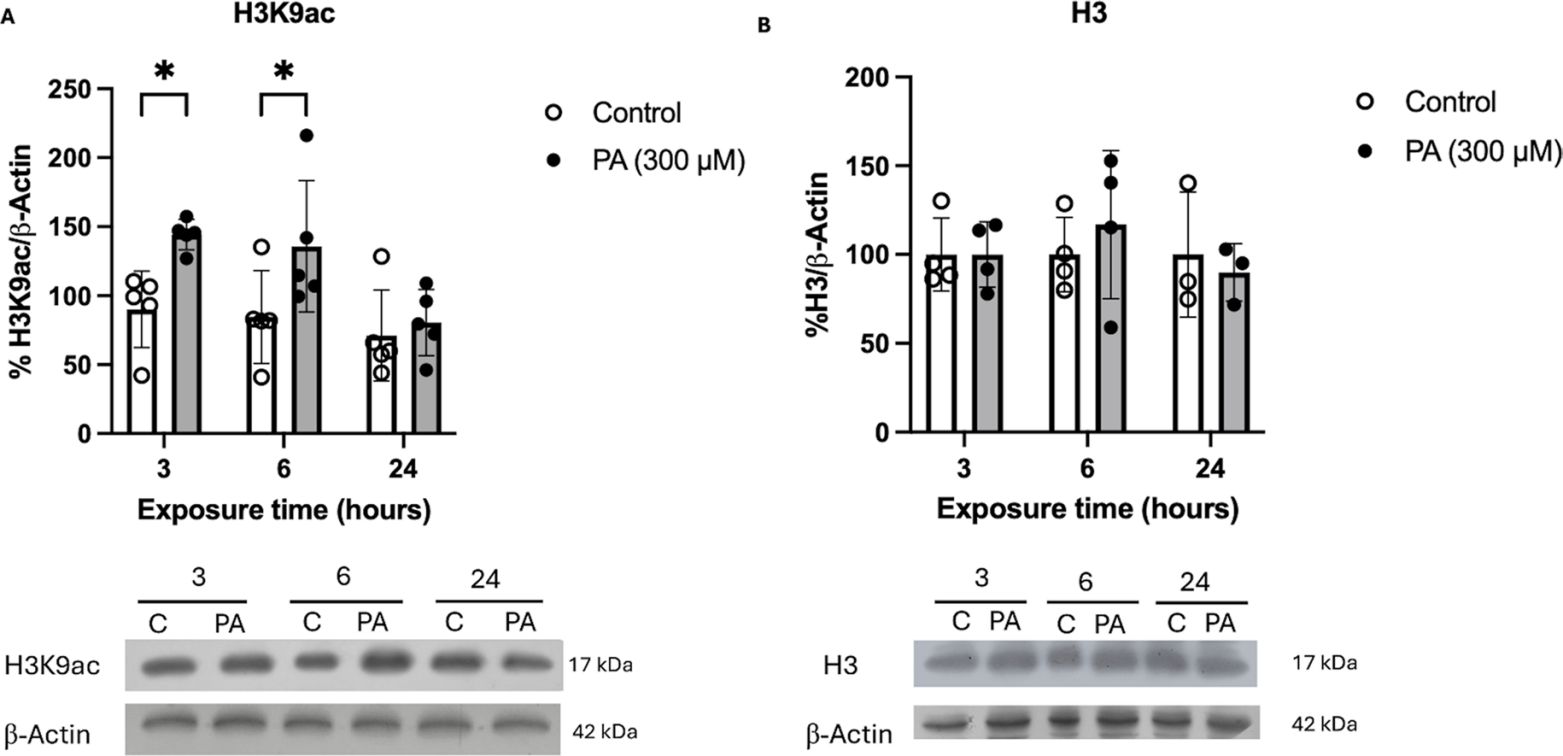
H3K9ac content increases after PA exposure without modifying total H3. (**A**) Densitometric analysis and representative western blot of H3K9ac. H3K9ac concentration increases after 3 (*p = 0.0359) and 6 h (*p = 0.0470) compared to the control group. No differences were observed at 24 h of PA treatment. Analyzed by 2-way ANOVA. (*n* = 5.) Results shown are the mean ± S.E.M. of 5 independent experiments. (**B**) Densitometric analysis and representative western blot of total H3. No changes are observed at any exposure time between the treated and control groups. Analyzed by two-way ANOVA (*n* ≥ 3). Results shown are the mean ± S.E.M. of at least 3 independent experiments

In light of the observed dynamic changes in HDAC2 and HDAC3 levels, we aimed to examine if these alterations correlated with changes in the acetylation levels of the lysine 9 of histone H3 (H3K9ac). The results revealed that the H3K9ac content increased after 3 and 6 h following a PA treatment but returned to control levels at 24 h (Fig. 3A). No difference was observed in the total content of H3 (Fig. 3B).

**Fig. 4:**
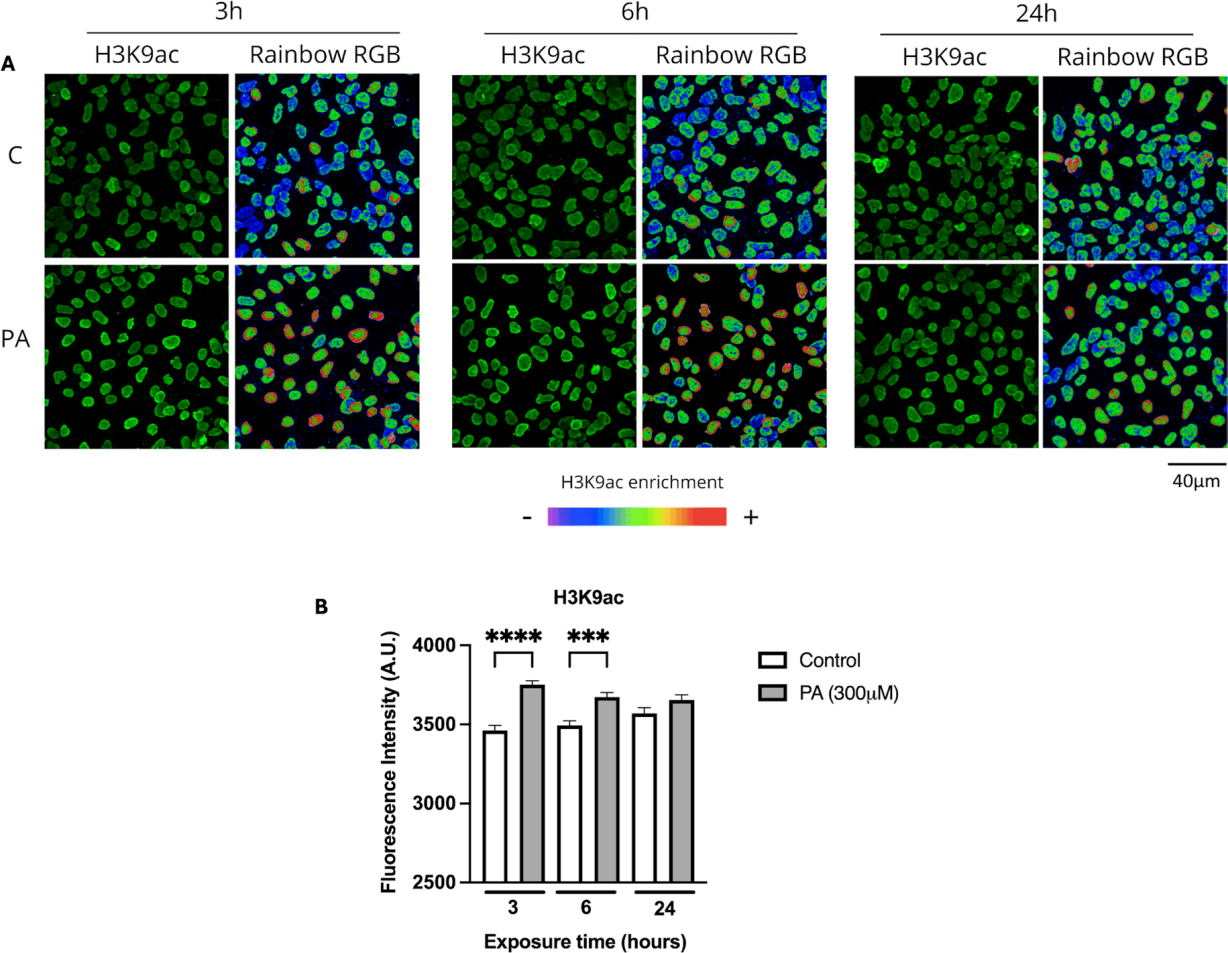
H3K9ac fluorescence immunodetection. (**A**) Representative images of the maximum intensity z-stack projection of H3K9ac after a PA treatment (3, 6, and 24 h) acquired by confocal microscopy. H3K9ac is shown in green, and its enrichment is shown with a Rainbow RGB color filter. (**B**) Quantification of the maximum intensity of the z-projection of H3K9ac. At 3 h, H3K9ac increases compared to its respective control (****p = < 0.0001), it remains increased at 6 h, although to a lesser extent than at 3 h (p=***0.0005), and finally it returns to levels similar to those of the control group at 24 h (100 nuclei per condition from 3 independent biological replicates). Analyzed by 2-way ANOVA. The results shown are the mean ± S.E.M. of the arbitrarily count of 100 nuclei per experimental condition from 3 independent biological replicates

To further confirm the observed results of the H3K9ac increase and to characterize its localization, fluorescence immunodetection and confocal microscopy analyses were performed following the treatment with 300 µM PA at 3, 6, and 24 h of PA exposure (Fig. 4A. After 3 h of PA there was a significant increase of 7.7% in H3K9ac compared to the control condition, and it remained elevated until 6 h although at lower levels (4.92%). Finally, at 24 h there was no statistically significant change between the experimental and control condition (Fig. 4B).

**Fig. 5:**
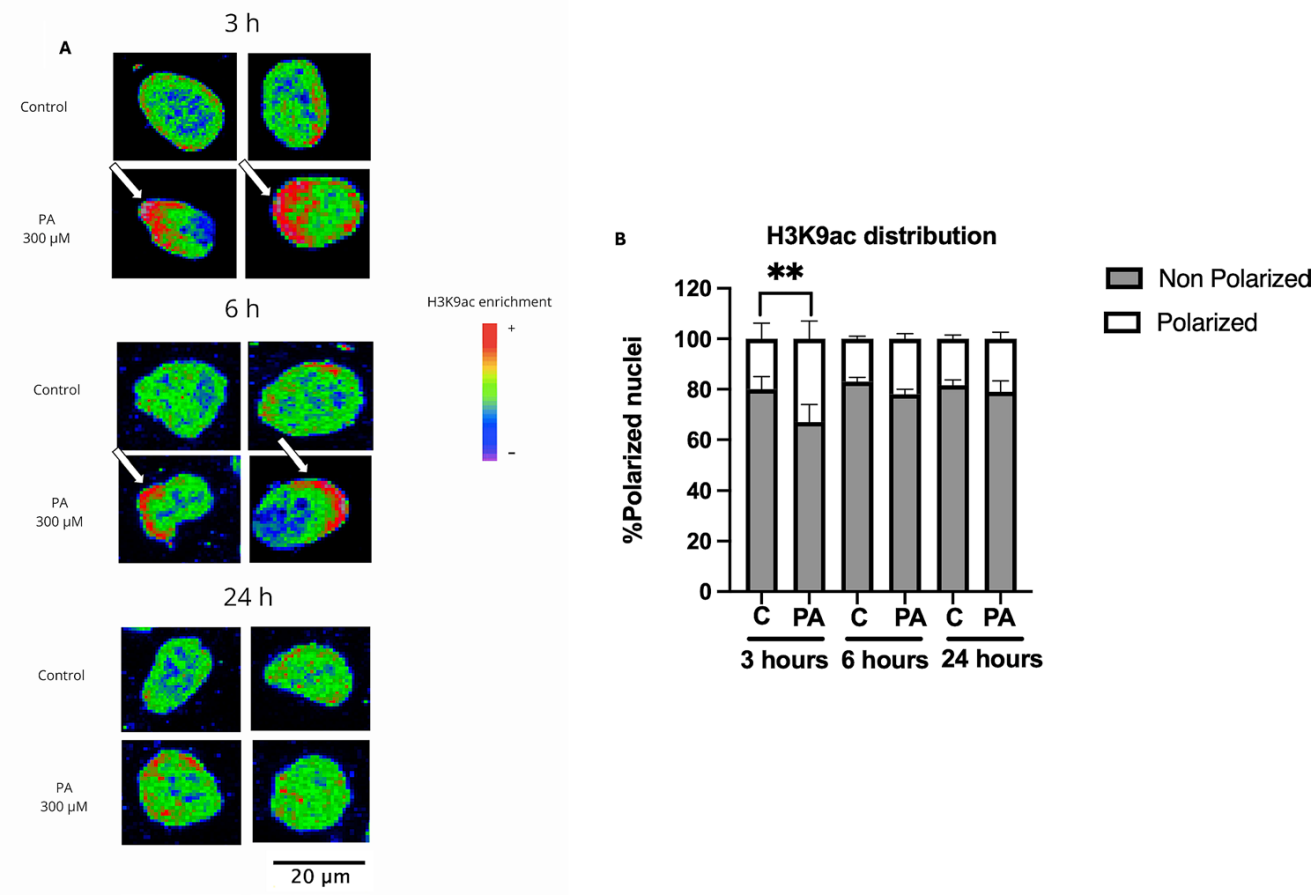
H3K9ac changes its nuclear distribution following a short-term PA exposure. (**A**) Representative images of the fluorescence immunodetection of H3K9ac after 3, 6, and 24 h PA treatment using maximum intensity of a z-stack with a Rainbow RGB filter. Increased magnification of nuclei is shown. The white arrows point the polarization of H3K9ac, mainly located at one side of the nucleus. (**B**) Quantification of the polarization of H3K9ac. All groups showed polarized nuclei. However, a higher number of polarized nuclei is observed at 3 h of PA exposure compared to control (**p = 0.0023). At 6 h a less pronounced increase was observed (*p* = 0.394) and at 24 h the difference between groups was minimal (100 nuclei per condition of 3 independent biological replicates). Quantification in percentage of nuclei with a change in H3K9ac distribution. Analyzed by 2-way ANOVA. Results shown are the mean ± S.E.M. of the random count of 100 nuclei per experimental condition from 3 independent biological replicates

It is known that epigenetic post-translational modifications (PTM) cannot only increase or decrease in levels but also be redistributed within chromatin and throughout the nucleus [34, 35]. H3K9ac is an epigenetic PTM primarily located in euchromatic regions closer to the center of the nucleus. However, under stress conditions histone acetylation is able to shift from the center to the nuclear periphery [36]. Hence, we analyzed if H3K9ac was polarized in PA-exposed neurons by scoring the nuclei that showed the H3K9ac fluorescence intensity in the center or towards the nuclear periphery. The analysis showed that under control conditions, as expected, H3K9ac localized mainly in the center of the cell nucleus. Interestingly, some nuclei with polarized H3K9ac were observed under physiological conditions (Fig. 5A). However, after 3 h of treatment with 300 µM PA, the number of cells showing a significant polarized H3K9ac mark increased by approximately 15% compared to the control group. After 6 h of treatment, the number of cells with H3K9ac redistribution remained increased (5%) compared to the control condition but it was not statistically significant while showing a tendency and finally remained similar to control levels after 24 h of PA treatment (Fig. 5B). These results indicate that PA-dependent increase of H3K9ac is sufficient to cause dynamic changes in its nuclear distribution after short-term exposure.

### Coding and Non-Coding Gene Expression are Dynamically Regulated in a Time-Dependent Manner After PA Exposure

**Fig. 6:**
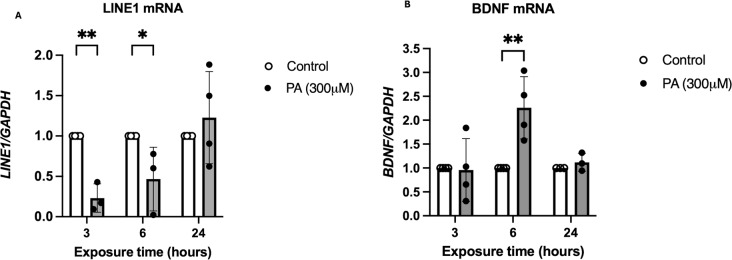
Repeated elements and BDNF expression is differentially modulated in a time-dependent manner following a PA treatment. (**A**) LINE1 mRNA quantification. The transcription is diminished drastically at 3 h of PA exposure (**p = 0.0032) and appears to recover through time (*p = 0.0286) reaching control levels at 24 h post-treatment. Analyzed by 2-way ANOVA. Results shown are the mean ± S.E.M. of 3–4 independent experiments (**B**) BDNF mRNA quantification. There is an increase at 6 h post-treatment (**p = 0.0051). Analyzed by 2-way ANOVA. Results shown are the mean ± S.E.M. of 3–4 independent experiments

As it was previously reported that HDAC2 and HDAC3, through histone deacetylation, are able to modulate the expression of genes associated with plasticity, synaptic transmission, and neuronal survival, such as *BDNF* [37–40], and that histone acetylation plays a role in the regulation of *LINE1* transcription [41–43], we sought to compare the expression of these coding and non-coding genes after PA treatment to correlate it with H3K9ac levels (Fig. 6). We observed that in response to PA treatment, the expression of the transposable element *LINE1* was severally reduced after 3 h PA exposure compared to control condition (Fig. 6A). The same effect was observed when the expression of the Satellite 2 was evaluated at 3 h of PA exposure (data not shown). After 6 h of PA treatment the expression of *LINE1* is still reduced and at 24 h returned to control levels compared to the control condition (Fig. 6A). Conversely, an evident expression of *BDNF* was detected at 6 h post-PA treatment (Fig. 6B). Interestingly, the decrease of *LINE1* occurs simultaneously with the increase of HDAC3, and the increase in *BDNF* transcript coexists with increased H3K9ac and also coincides with a time point at which there are no changes in HDAC2 or HDAC3 contents. Overall, these data suggest that PA treatment might be associated with an alteration in the expression of coding and non-coding genes in a time-dependent manner, possibly related to mechanisms that modulate to the balance between lysine acetyltransferases (KATs) and HDACs, and therefore the increase of H3K9ac. Nevertheless, the action and content of KATs needs further validation to shed light on the role they are playing in these metabolic conditions.

## Discussion

The intake of PA increased considerably in recent years and has been shown to exert metabolic pleiotropic effects resulting in cellular homeostatic imbalance. However, the mechanisms by which neuronal physiology is altered by high PA concentrations, including changes in gene expression, remain largely unknown. In this study we evaluated the PA impact on some metabolic and epigenetic modifications that could influence gene expression. Histone acetylation/deacetylation is one of the proposed epigenetic mechanisms that is suggested as a key player in the observed changes in the neuronal transcriptional profile. Therefore, we sought to identify and characterize the content of some relevant proteins responsible for the histone deacetylation process.

First, we provide evidence that PA activates energy metabolism as indicated by increased β-HB levels. This ketone body likely arises from active PA β-oxidation, consistent with reports in non-neuronal models [44], and in cortical neurons under hypoxia and hypoglycemia [45]. In prior work, we showed that PA induces metabolic activation in differentiated neuroblastoma cells through enhanced β-oxidation [24]. Separately, we demonstrated PA’s ability to reduce the NAD^+^/NADH ratio in hippocampal neurons, reflecting redox imbalance [46]. Altogether, these findings support the ability of neurons to metabolize this fatty acid through an energy-coupled biosynthetic pathway, a process poorly described in neurons. However, despite the statistical significance of the β-HB increase, it remains unclear whether it is physiologically sufficient to function as a PTM (β-hydroxybutyrylation) or as an enzymatic inhibitor of HDACs.

SFAs can not only be used as energy molecules, but they can also be stored as energy reservoirs or captured inside specialized storage-organelles known as lipid droplets or lipid bodies to prevent lipotoxicity [46, 47]. Interestingly, we found that the exposure of differentiated MSN cells to PA generated an increase in lipid droplets content. Although the majority of lipid droplets are composed of neutral lipids, mainly triglycerides, their accumulation can increase under stress conditions, aging or high exposure to SFAs, as tested in this study. This neuronal response may help the cells to meet energy requirements or mitigate lipotoxicity [48, 49].

Another reported metabolic consequence of neuronal PA exposure is the reduction of Sirt1 levels [32]. Interestingly, we found that SIRT1 decreased only after 24 h of PA treatment, with no significant changes at 3, 6–12 h post treatment. SIRT1 is a NAD^+^ dependent HDAC and a well-established metabolic sensor that links cellular metabolic status to gene expression by regulating transcription factors [50–52]. Since PA not only had an effect on neuronal metabolism and lipidostasis but can also modify the transcriptional profile of different cell types [53, 54], it was particularly interesting that, in addition to SIRT1 reduction, nuclear class I HDACs were deregulated in a time-dependent manner. Considering that histone acetylation is highly dynamic and has a direct effect on chromatin conformation [55], HDACs dysregulation might be a potential epigenetic mechanism responsible for the changes in the transcriptional profile altered by PA. Our results showed that after 3 h of PA treatment, HDAC3 content was increased and occurred simultaneously with an unexpected increase of the H3K9ac content. This is an apparently contradictory result that may be explained through lipid energy metabolism. It was previously shown that neurons can β-oxidize PA, thus increasing acetyl-CoA production [10, 56–58]. This in turn, can serve as a substrate for KATs that may generate a hyperacetylated state of proteins including histones, as has been reported [58–60]. Hence, the high HDAC3 content could be due to a compensatory mechanism in order to counteract the H3K9ac levels, which effectively begins to decrease after a 6 h PA treatment. Conversely, HDAC2 was only overexpressed at 24 h after the PA treatment, which in turn, can explain the recovery of the basal-like levels of H3K9ac at 24 h. Overall, the results show the importance of continue evaluating the epigenetic machinery since all these complex mechanisms might act together as a neuronal response to a metabolic challenge [61, 62].

It is known that distribution of PTMs of histones, such as acetylation, can also be modified by stress, aging and in several pathological cellular states, altering the chromatin structure and gene expression [63–65]. The results in the present work showed that 3 h of PA exposure were sufficient to generate changes in the distribution of H3K9ac, polarizing the mark to one end of the nucleus. This mislocalization is still present after 6 h of PA treatment, although without statistical significance, and returns almost fully to baseline-like levels after 24 h of the metabolic stimuli. It is worth noting that the H3K9ac content was also increased after 3 and 6 h of PA treatment, making it possible that this redistribution is closely related to the H3K9 hyperacetylated state. Recent evidence demonstrates that in mouse embryonic fibroblast nuclei, an increased number of nuclear deformations called “nuclear blebs” are generated when euchromatic regions are concentrated in the nuclear periphery, specifically co-localizing with H3K9ac-rich areas [36]. This evidence supports our hypothesis of how the increased H3K9ac content is related to euchromatin redistribution and the possible effects it could have on nuclear morphology. This epigenetic PTM is characteristic of euchromatin [66], it is present in the promoter region of active genes [67], and is tightly associated with increased gene expression [66]. Thus, changes in the content of this PTM can have a direct impact on gene transcription [68]. Although we did not observe the formation of “nuclear blebs” in any of the examined fields, the significant redistribution of the H3K9ac suggests a previous step of stronger changes in the nuclear morphology induced by PA exposure. However, when gene expression was quantified, we observed that *BDNF* levels increased transiently after 6 h of PA treatment, time at which there was an increased content of H3K9ac. Chromatin immunoprecipitation analysis have demonstrated that *BDNF* expression can be regulated by H3K9ac [37, 69], suggesting that the observed increase in BDNF expression might be associated with the hyperacetylated H3K9 [70]. Interestingly, at 3 and 24 h post PA treatment there was no increase in *BDNF* expression compared to the control group. Notably, these time points of PA exposure coincided with an increase in HDAC3 (at 3 h) and HDAC2 (at 24 h) expression, respectively. In this context, it seems that high levels of HDAC2 and HDAC3 are related to lower *BDNF* expression [38, 71] highlighting the complex dynamic interplay between HDACs, H3K9ac, and *BDNF*.

Conversely to what we expected, the expression of the non-coding gene LINE1 behaved different to *BDNF* since its expression was drastically reduced after 3 h of PA and gradually reestablishes until it reaches the control levels at 24 h. Given that LINE1 is known to respond to alterations in histone acetylation and repressive complexes where HDAC3 is part of, such as the NCoR/SMRT [41, 42, 72], hence, it is plausible that the upregulation of HDAC3 at 3 h contributes to LINE1 repression, potentially counteracting the effects of global H3K9 hyperacetylation. Nevertheless, this hypothesis needs further research.

**Fig. 7:**
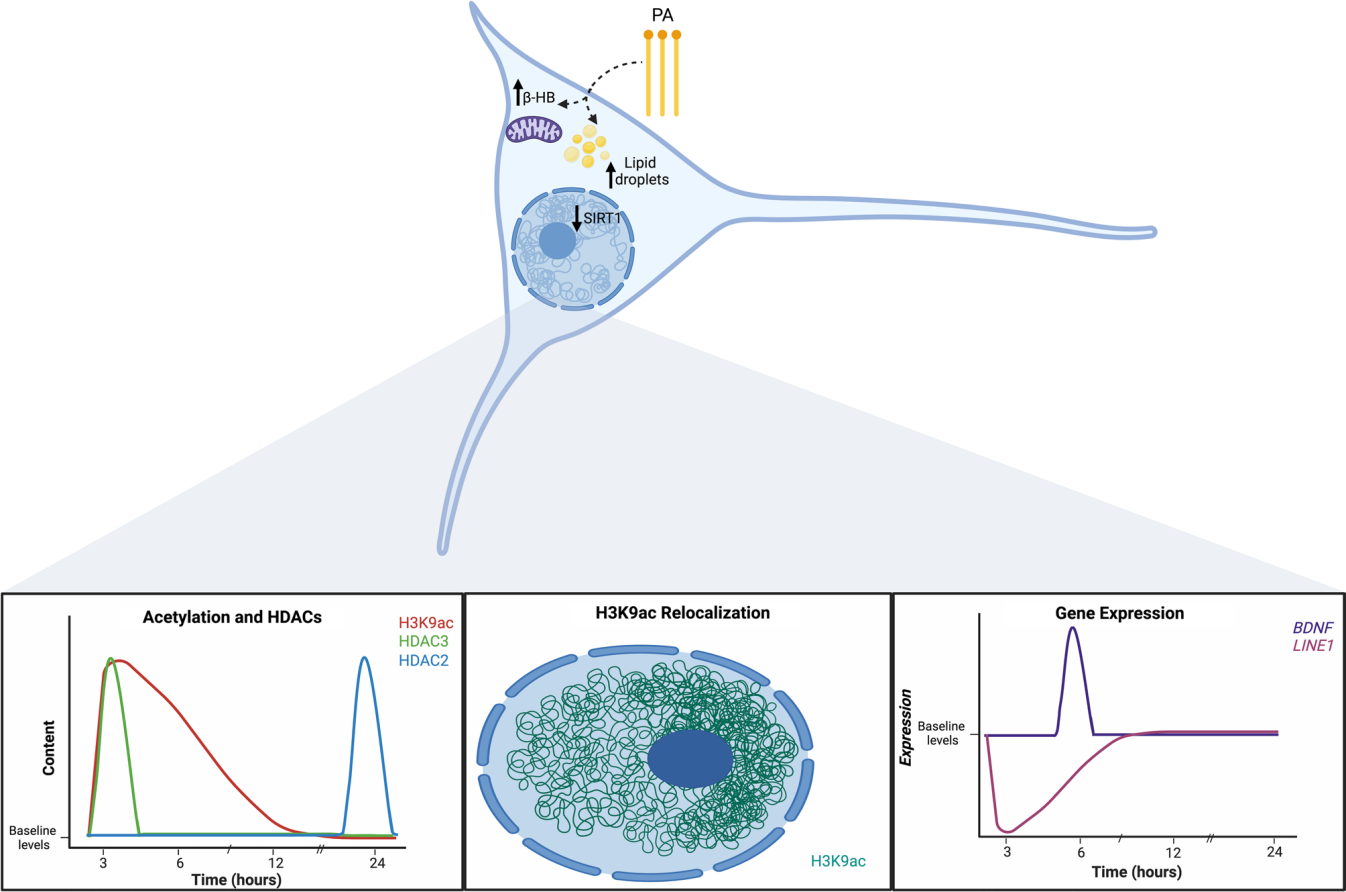
Neurons are capable of metabolizing PA and the exposure to this SFA generates time-dependent changes in epigenetic-associated events. PA metabolism increases lipid droplets, and the synthesis of β-HB. Furthermore, the metabolic sensor SIRT1 content is decreased. In a time-dependent manner, after short-time treatments (3 h) a rise in H3K9ac levels occurs, accompanied by a polarization of this PTM to one side of the nucleus, and an overexpression of class I HDAC, HDAC3. Also, after 3 h, the repeated sequence *LINE1* shows a profound decrease. After 6 h, H3K9ac levels remain increased, associated with the overexpression of *BDNF*. And *LINE1* levels remain decreased but to a lesser extent than after a 3-hour exposure. Finally, after a long-time treatment (24 h), H3K9ac levels return to baseline levels, this might be associated with the increased content of HDAC2 after 24 h of a PA treatment

Together these findings suggest that PA exposure induces time-dependent and gene-specific epigenetic responses in neurons. The transient increase in *BDNF* may reflect early adaptive response linked to metabolic changes, while the later repression of *BDNF* and LINE1 appears to be mediated by the upregulation of HDAC2 and HDAC3. Our results suggest that PA metabolism affects neuronal physiology by modulating both metabolism and some of the epigenetic machinery, resulting in differential regulation of coding and non-coding genes in a time-dependent manner (Fig. 7).

Finally, it should be noted that, to our knowledge, this is the first report providing insight into potential mechanisms affected following PA exposure that link neuronal energy metabolism, histone acetylation, chromatin structure, and the expression of a gene involved in neuronal plasticity, *BDNF* as well as a repetitive element important for genome stability and structure, *LINE1*.

## Data Availability

No datasets were generated or analysed during the current study.

## Abbreviations

HFD: High fat diets
PA: Palmitic acid
HDACs: Histone deacetylases
SFA: Saturated fatty acids
AD: Alzheimer’s disease
Sirt1: Sirtuin 1
H3K9ac: Acetylation of the lysine 9 of histone H3
PTM: Post-translational modifications
LINE1: Long interspersed element-1
BDNF: Brain-derived neurotrophic factor
KATs: Lysine acetyltransferases
